# Changing Demographics of Aging and Disability: Implications for Advancing Knowledge of Disablement and Life Course

**DOI:** 10.1093/geroni/igaa057.2213

**Published:** 2020-12-16

**Authors:** Margaret Campbell

**Affiliations:** Campbell & Associates Consulting, Grapeview, Washington, United States

## Abstract

Increased survivorship and longevity have resulted in dramatic improvements in life quality for people with significant disabilities and impairments. However, the fields of rehabilitation and gerontology have tended to divide this phenomenon into people aged under 65 aging with lifelong and early onset disabilities, and those aged 65 plus who are aging into late onset disability. But for both groups, increased survivorship also translates into more years living with comorbidities associated with the underlying condition and increased risk for premature onset and higher rates of age-related chronic conditions. Despite these widely acknowledged trends, we have no national data systems that estimate the overall prevalence of the ‘aging with long-term disability’ population and monitor its status. Acknowledged is that the lack of national data and reliance on chronological age undermines our knowledge of the disablement experience across the life course and the needs for services and supports associated with diverse trajectories. Part of a symposium sponsored by the Lifelong Disabilities Interest Group.

